# Supplementary material for: A high-lipid diet leads to greater pathology and lower tolerance during infection
Source: J Exp Biol. 2025 Mar 5;228(5):JEB249541. doi: 10.1242/jeb.249541 (PMC11925396; doi:10.1242/jeb.249541)
Supplement: Supplementary information [file jexbio-228-249541-s1.pdf]

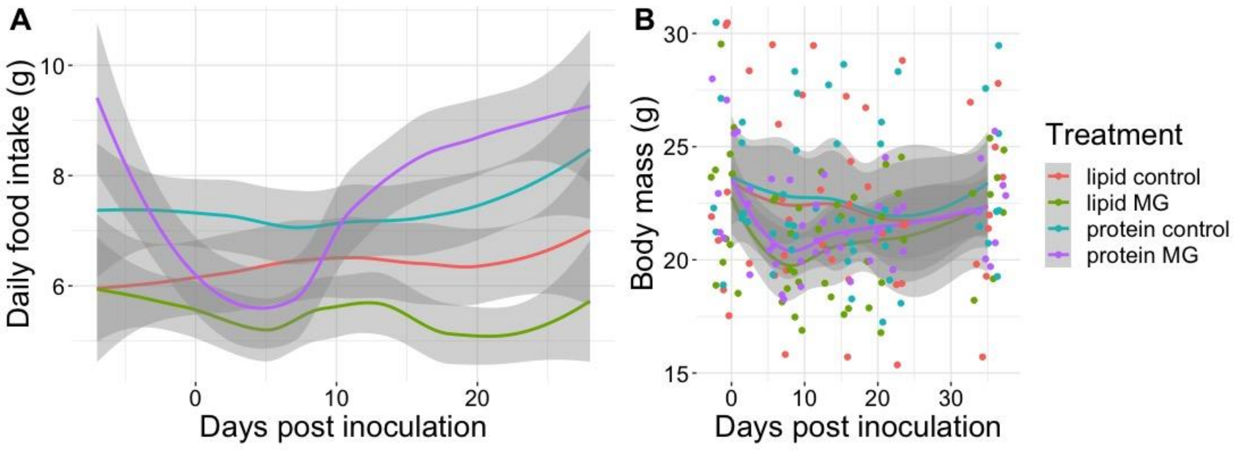

Fig. S1. Effect of diet (lipid or protein) and *Mycoplasma gallisepticum* (MG) or sham inoculation on A) daily food intake (g) and B) body mass (g) of canaries (*Serinus canaria domestica*) over time in experiment one. Points represent raw data with smoothed average trend lines surrounded by 95% confidence interval bands in grey.

Journal of Experimental Biology • Supplementary information

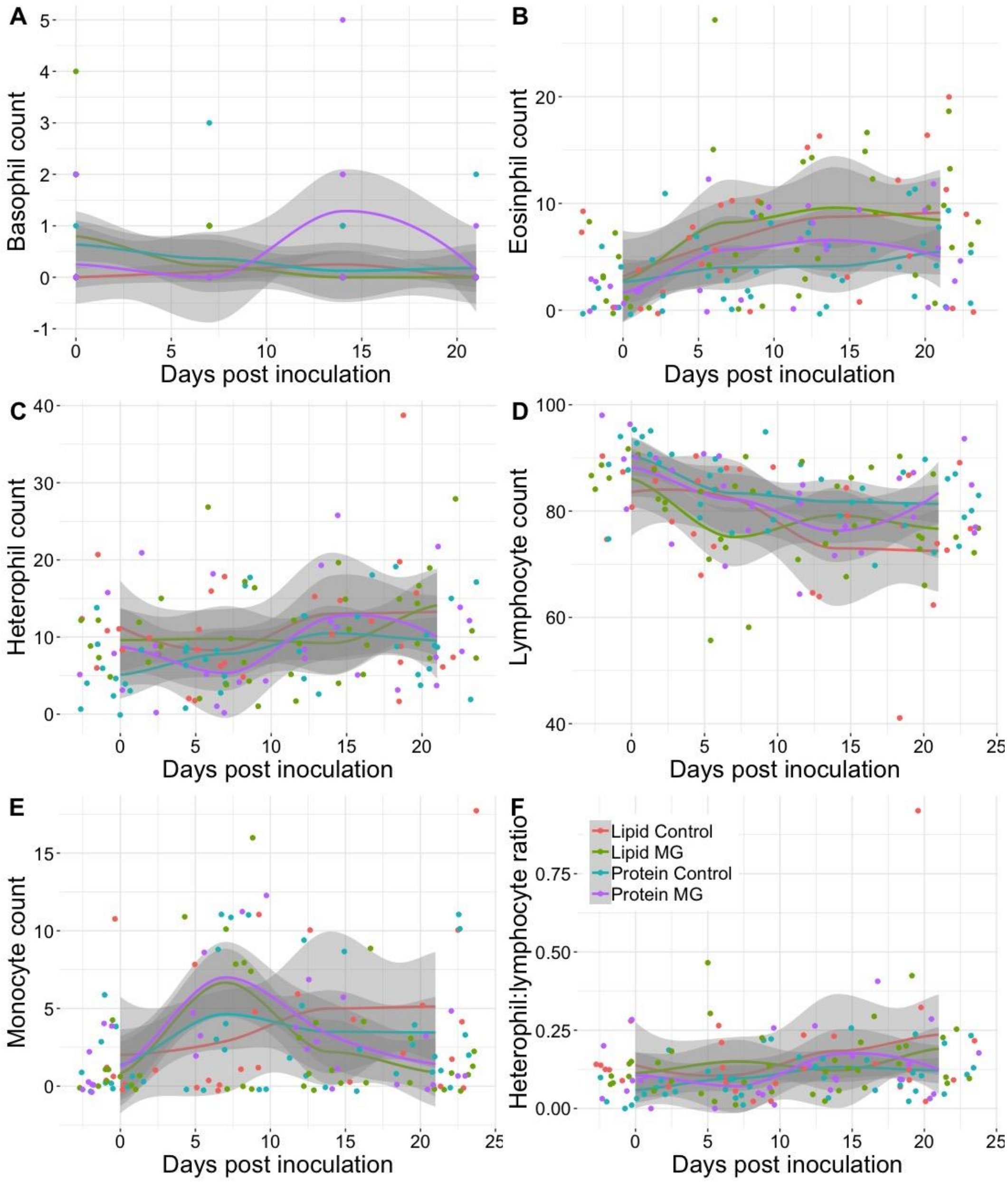

Fig. S2. Effect of diet (lipid or protein) and *Mycoplasma gallisepticum* (MG) or sham inoculation on relative abundance of white blood cells over time from canaries (*Serinus canaria domestica*) in experiment one. Points represent raw data with smoothed average trend lines surrounded by 95% confidence interval bands in grey.

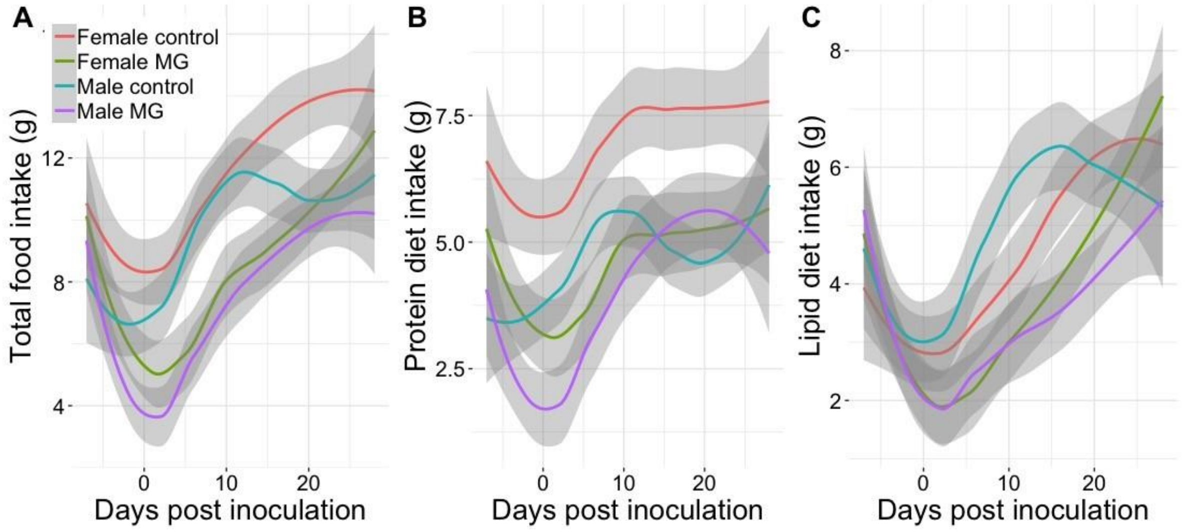

Fig. S3. Effect of sex and *Mycoplasma gallisepticum* (MG) or sham inoculation on A) total, B) protein, and C) lipid daily food of canaries (*Serinus canaria domestica*) intake over time during experiment two. Average trend lines are surrounded by 95% confidence interval bands in grey.

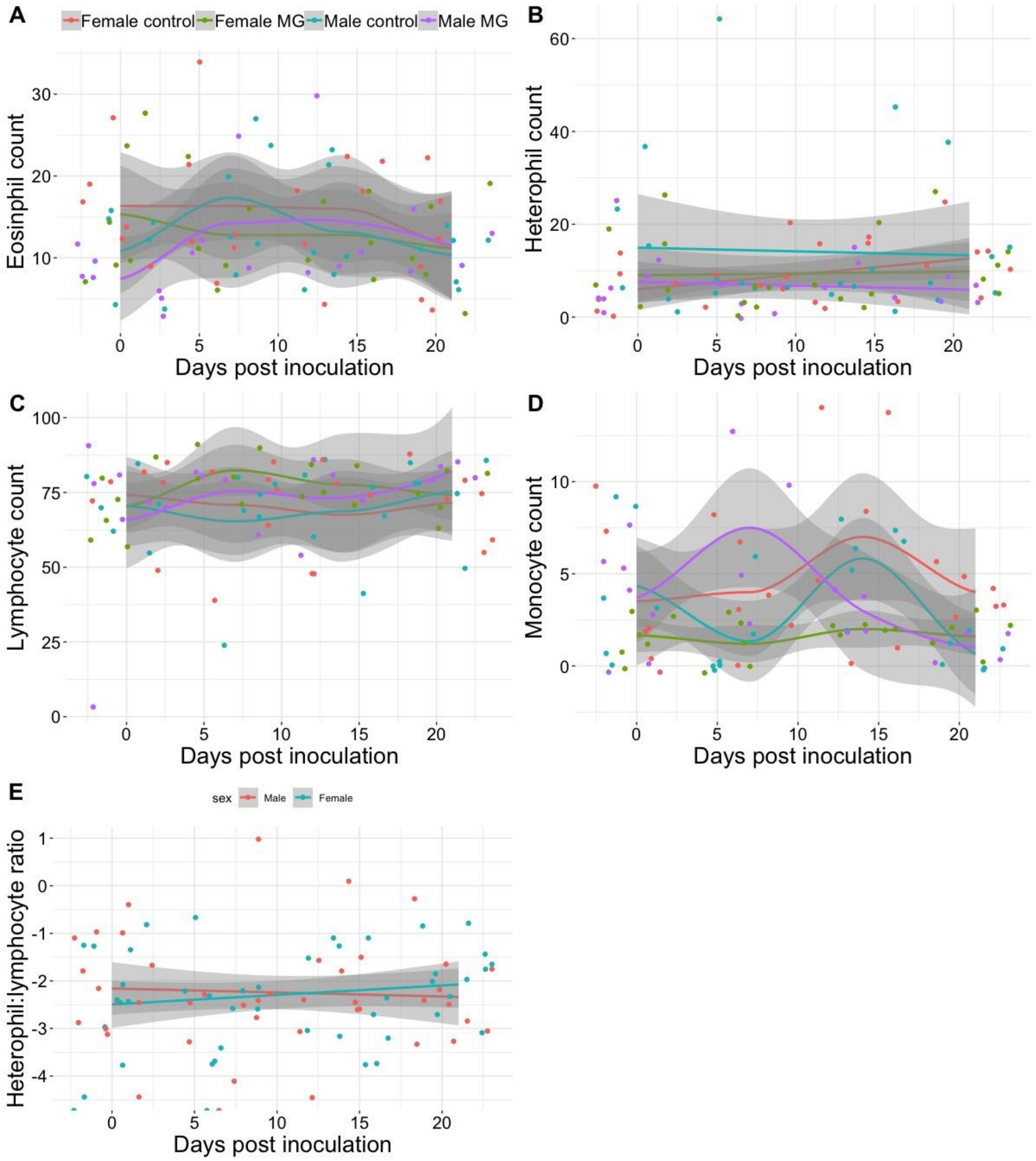

Fig. S4. Relative abundance of white blood cells over time from canaries (*Serinus canaria domestica*) in experiment one. Points represent raw data with average trend lines surrounded by 95% confidence interval bands in grey. MG is short for *Mycoplasma gallisepticum.*

Journal of Experimental Biology • Supplementary information
